# Highly Selective Bioconversion of Ginsenoside Rb1 to Compound K by the Mycelium of *Cordyceps sinensis* under Optimized Conditions

**DOI:** 10.3390/molecules201019291

**Published:** 2015-10-23

**Authors:** Wei-Nan Wang, Bing-Xiong Yan, Wen-Di Xu, Ye Qiu, Yun-Long Guo, Zhi-Dong Qiu

**Affiliations:** School of Pharmaceutical Sciences, Changchun University of Chinese Medicine, Changchun 130117, China; E-Mails: cnweinanwang@163.com (W.-N.W.); ybx10528@163.com (B.-X.Y.); 15584452505@163.com (W.-D.X.); ccqy19890810@163.com (Y.Q.); ccyunlong1016@126.com (Y.-L.G.)

**Keywords:** *Cordyceps sinensis*, ginsenoside Rb1, compound K, biotransformation, optimization

## Abstract

Compound K (CK), a highly active and bioavailable derivative obtained from protopanaxadiol ginsenosides, displays a wide variety of pharmacological properties, especially antitumor activity. However, the inadequacy of natural sources limits its application in the pharmaceutical industry. In this study, we firstly discovered that *Cordyceps sinensis* was a potent biocatalyst for the biotransformation of ginsenoside Rb1 into CK. After a series of investigations on the biotransformation parameters, an optimal composition of the biotransformation culture was found to be lactose, soybean powder and MgSO_4_ without controlling the pH. Also, an optimum temperature of 30 °C for the biotransformation process was suggested in a range of 25 °C–50 °C. Then, a biotransformation pathway of Rb1 → Rd → F2 → CK was established using high performance liquid chromatography/quadrupole time-of-flight mass spectrometry (HPLC-Q-TOF-MS). Our results demonstrated that the molar bioconversion rate of Rb1 to CK was more than 82% and the purity of CK produced by *C. sinensis* under the optimized conditions was more than 91%. In conclusion, the combination of *C. sinensis* and the optimized conditions is applicable for the industrial preparation of CK for medicinal purposes.

## 1. Introduction

*Panax ginseng* C. A. Meyer is a widely used oriental herb with multiple clinical and pharmacological effects related to cancer, immunodeficiency and diabetes mellitus, *etc.* [1,2,3,4]. The saponins contained in *Panax ginseng*, named ginsenosides, are proven to be the major bioactive components (Table 1). However rare ginsenosides (Rg3, F2, Rh1, Rh2, CK, *etc.*) present in low concentration or absent in *Panax ginseng* often have better performances in bioactivity and bioavailability than others [5,6,7,8,9,10]. Hence many research works have been done over the past decades on how to increase the quantities of these rare ginsenosides.

**Table 1:** Chemical structure of major and rare ginsenosides in *Panax ginseng*.

**20(*S*)-Protopanaxdiol Type**				
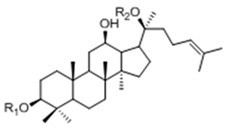	Ginsenoside	R1 (C-3)		R2 (C-20)
	Rb1	-Glc2-Glc		-Glc6-Glc
	Rd	-Glc2-Glc		-Glc
	Rg3	-Glc2-Glc		-H
	F2	-Glc		-Glc
	Rh2	-Glc		-H
	Compound K	-H		-Glc
**20(*S*)-Protopanaxtriol Type**				
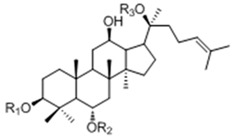	Ginsenoside	R1 (C-3)	R2 (C-6)	R3 (C-20)
	Re	-H	-Glc2-Rha	-Glc
	Rf	-H	-Glc2-Glc	-H
	Rg1	-H	-Glc	-Glc
	Rg2	-H	-Glc2-Rha	-H
	Rh1	-H	-Glc	-H

Theoretically, these ginsenosides can be obtained by either direct synthesis or by deglycosylating major ginsenosides (Rb1, Rd, Re, Rg1, *etc.*) through chemical approaches. Nevertheless, these approaches are usually impractical to obtain highly purified ginsenosides for their poor regiospecificity and concurrent hydrolysis. In that case, biotransformations, which are considered to be highly efficient and regiospecific processes, might be a promising method [11,12,13,14,15]. There are two sound ways to accomplish the biotransformation of ginsenosides: microbial transformation and enzymatic biotransformation. Although the production of CK from other protopanaxadiol-type ginsenosides has been achieved via enzymatic methods using β-glucosidase, β-galactosidase, lactase and pectinase, *etc.*, limitations on industrialization are still challenging problems for the application of enzymatic biotransformations [16].

Microbial transformations, on the other hand, have advantages over purified enzymes in industrial fermentation, even if the permeability barrier of the cell envelope for substrates and products often causes very low reaction rates in whole cells [17]. Thus, screening for microorganisms with high catalytic specificity and biotransformation efficacy is crucial for the industrial production of rare ginsenosides. Bae *et al*. discovered that *Bacteroides* sp., *Eubacterium* sp. and *Bifidobacterium* sp. isolated from human fecal microflora could potently transform ginsenoside Rc into CK [18]. Zhou *et al*. applied a fungal system instead of intestinal bacteria to biotransform *Panax notoginseng* saponins (PNS) resulting in a much higher production of CK than those described in previous reports [19]. More fungi like *A. niger*, *A. usamii* [20], *A. strictum* [21] and *F. moniliforme* [22] were proven to be efficient in biotransforming certain ginsenosides into CK. Taken together, these references suggest microbial transformations should be a promising way to obtain CK in abundance.

*Cordyceps sinensis* is a valuable medicinal fungus with a long history of safe use as a general tonic and an aphrodisiac medicine [23,24,25,26]. It has good reputation for genetic stability, environmental adaptation and versatile metabolic enzymes, which make it a potentially excellent biocatalyst for many substrates [27,28]. However, there are few references published till now regarding the biotransformation characteristics of *C. sinensis*.

In the present study, we have screened *C. sinensis* from several well-known medicinal fungi for their biotransformation abilities towards ginsenoside Rb1. After we conducted a series of optimizations on the fermentation parameters, including carbon source, nitrogen source, biotransformation elicitors, fermentation pH and biocatalytic temperature, CK had been efficiently produced in high yields. In addition, interesting results were unveiled during the optimizations, leading us to a deeper insight on the biotransformation of ginsenosides.

## 2. Results and Discussion

### 2.1. Optimization on Biotransformation Parameters of Ginsenoside Rb1 by C. sinensis

#### 2.1.1. Determination on Substrate-Feeding Time

**Figure 1:**
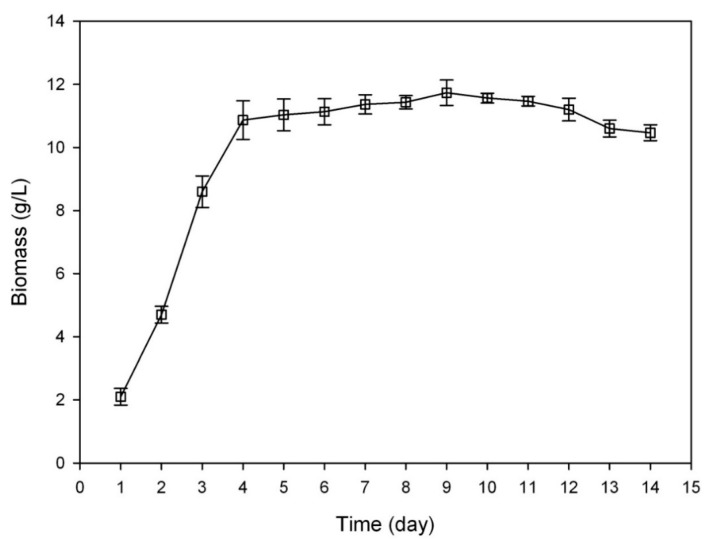
The growth curve of *C. sinensis* grown on basal medium (BM). The error bars indicate standard deviations of the dry weight.

Microorganisms in different growth phase usually show different enzymatic potency. Thus it is a prerequisite for any fermentation industry to explore the growth curve of the microbes in use. In this study, we measured the time-dependent changes in the dry weight of *C. sinensis*. The growth curve is depicted in Figure 1. It appeared that *C. sinensis* reached its exponential growth phase after one day of adaptation to the new environment and the accumulation of biomass was quickly completed at day 5, reaching its stationary phase. Thereafter, it stayed at this phase for nearly 8 days until the dry weight begin to decrease at day 12, entering its decline phase.

Microorganisms often need more nutrients to meet the requirements for proliferating at a rapid pace. Therefore, more catabolic enzymes should be secreted to break down macromolecules in the cultures of microbes for a better digestion at the exponential growth phase. In that case, ginsenoside Rb1, which served as a donor of glucosyl residues, might be degraded by the same catabolic enzymes for the production of glucose. That was why we picked the *C. sinensis* cultures at day 1, day 2, day 3, day 4 and day 5, which were within the exponential phase, as biotransformation cultures to determine the proper substrate-feeding time.

**Figure 2:**
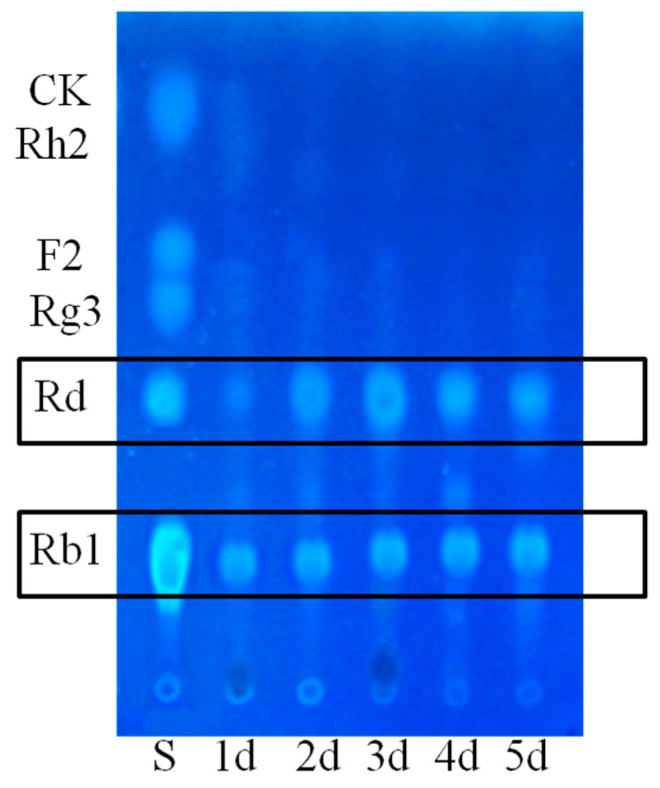
Influences of different substrate-feeding time on the biotransformation of Rb1.

As we can see in Figure 2, culture-dependant transformation of Rb1 to Rd (1) was apparent on days 2–5, but the production of Rd (1) from 1-day-old culture was not obvious. Although there was no apparent difference in the biotransformation performances between the cultures from day 2 to day 5, the liquid culture of *C. sinensis* would gradually become mucous along with the accumulation of exogenous metabolites such as polysaccharides and proteins, leading to complex conditions for further processing. Besides, preparing older cultures could be time-consuming, suggesting that the 2-day-old culture of *C. sinensis* was more suitable than the other three cultures of day 3, day 4 and day 5.

#### 2.1.2. Optimization on Carbon Sources, Nitrogen Sources, Elicitors, Reaction pH and Temperature for the Biotransformation Process

Carbon source and nitrogen source are two essential components for the growth and metabolism of microorganisms. They were proven to induce the expression of some enzymes [29,30] and supposed, in this case, to affect the biotransformation ability of *C. sinensis*. Hence, we chose some carbon sources and nitrogen sources to investigate their influences on the biotransformation of ginsenoside Rb1.

**Figure 3:**
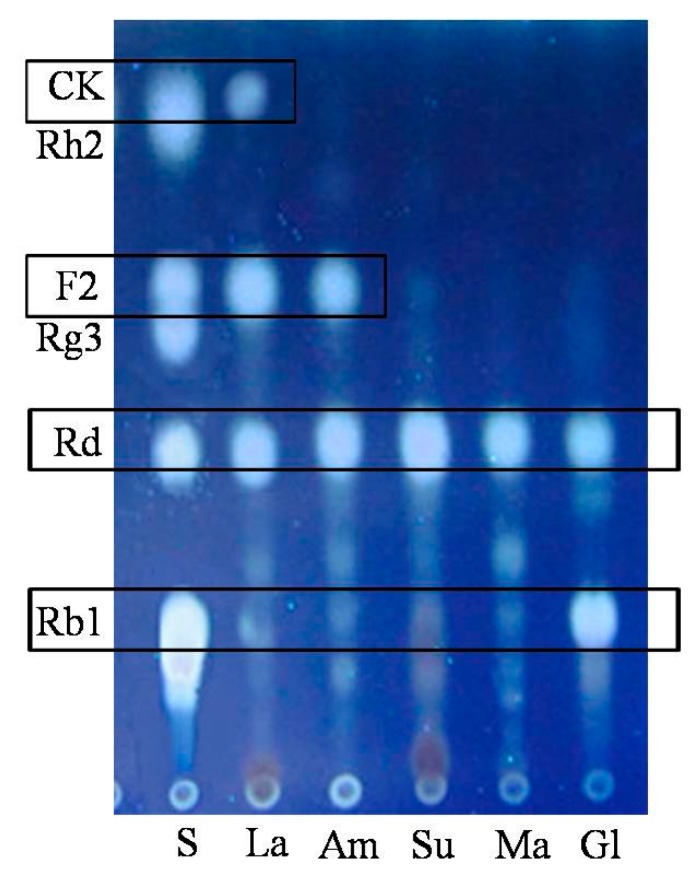
Influences of different carbon sources on the biotransformation of Rb1. La: lactose; Am: amidulin; Su: sucrose; Ma: maltose; Gl: glucose.

As depicted in Figure 3, *C. sinensis* in the lactose group could biotransform Rb1 into Rd (1), F2 (2) and CK (3) after 4 days of fermentation, while in glucose group, only one glucosyl moiety at C-20 of ginsenoside Rb1 had been hydrolyzed. We hypothesized that fewer glycosidases were secreted while glucose could be easily ingested by *C. sinensis* as a primary form of energy. Moreover, this result was consistent with the reference that glucose might inhibit the activity of some glycosidases due to a putative substrate inhibition mechanism, provided that glucose was one of the products from the biotransformation of Rb1 [31]. In total, the contribution order of different carbon sources to the biotransformation of ginsenoside Rb1 was lactose > amidulin > sucrose/maltose > glucose judging from their TLC performances.

**Figure 4:**
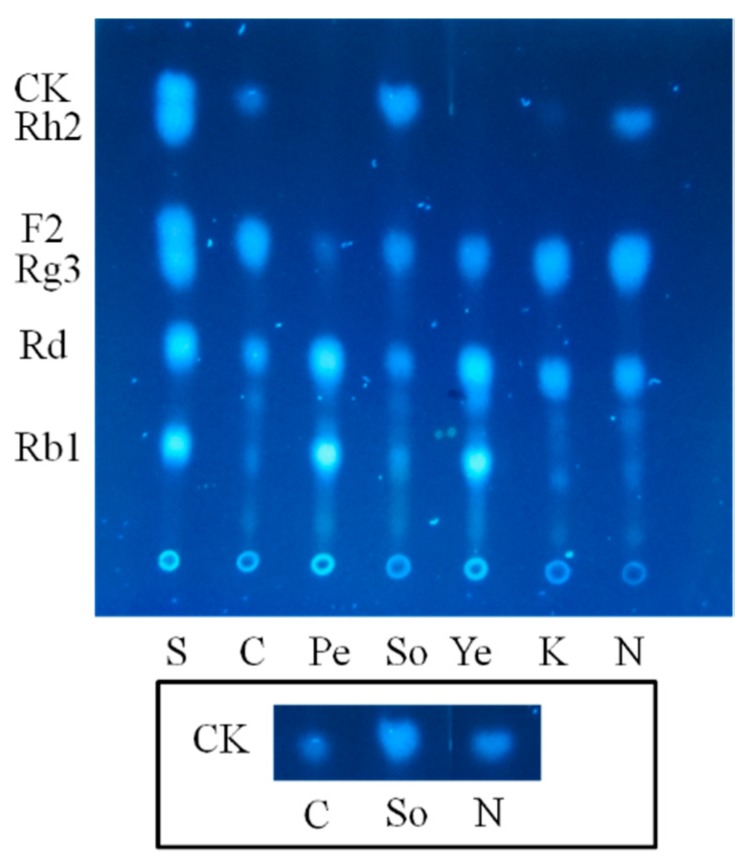
Influences of different nitrogen sources on the biotransformation of Rb1. C: control; Pe: peptone; So: Soybean powder; Ye: yeast; K: KNO_3_; N: (NH_4_)_2_SO_4_. The inlay demonstrates the TLC bands of CK from the groups of control, soybean powder and (NH_4_)_2_SO_4_.

In order to accelerate the reaction, we further optimized the nitrogen sources which are major suppliers of building blocks for the synthesis of proteins and nucleosides that prominently influence the accumulation of biomass and production of enzymes. Either organic or inorganic sources were employed with the lactose as carbon source to comprehensively screen the optimum nitrogen sources. Unexpectedly, the entire group of peptone, yeast powder and KNO_3_ hampered lactose for converting ginsenoside F2 (2) into CK (3). In contrast, the lactose-only group (control), soybean powder group and (NH_4_)_2_SO_4_ group all further biotransformed ginsenoside F2 (2) into CK (3) after 4 days of fermentation. Although, there was no seeming difference in biotransformation process acceleration between these two nitrogen sources, soybean powder dominated for its higher production of CK (3) as quantified by TLC visualization (Figure 4).

**Figure 5:**
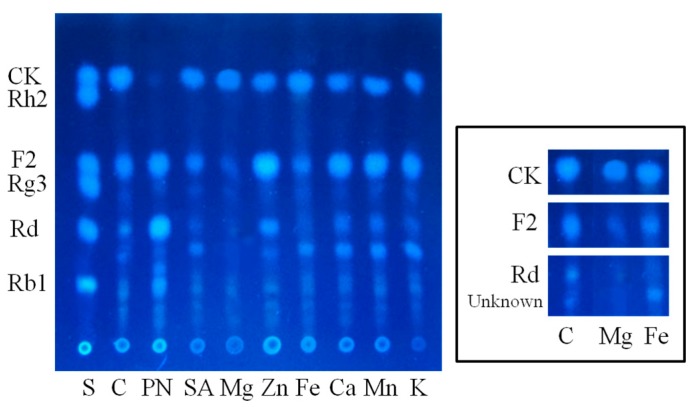
Influences of different elicitors on the biotransformation of Rb1. C: control; PN: PNPG; SA: sodium salicylate; Mg: MgSO_4_; Zn: ZnSO_4_; Fe: FeSO_4_; Ca: CaCl_2_; Mn: MnSO_4_; K: KH_2_PO_4_.

Since diverse glycosidases are supposed to participate in the biotransformation of ginsenoside Rb1, elicitors were screened in the lactose and soybean powder medium to increase the ability of target enzymes [31,32,33]. To avoid unnecessary chemical reactions between the selected elicitors and components in the medium, the elicitor solution was sterilized with an aseptic filter before mixing with the sterilized medium. Somehow, PNPG, widely used as glycosidase elicitor *in vitro*, hindered the biotransformation progress after ginsenoside F2 (2) was produced (Figure 5). Both MgSO_4_ and FeSO_4_ expressed significant glycosidase-inducing ability with *C. sinensis*. Nevertheless, the MgSO_4_ group had less byproduct after 4 days of fermentation and was more beneficial for the growth of *C. sinensis* than the FeSO_4_ group. Thus, an optimal composition of medium for the biotransformation of Rb1 to CK (3) was established as follow: 10 g/L soybean powder, 20 g/L lactose, 1 mM MgSO_4_.

**Figure 6:**
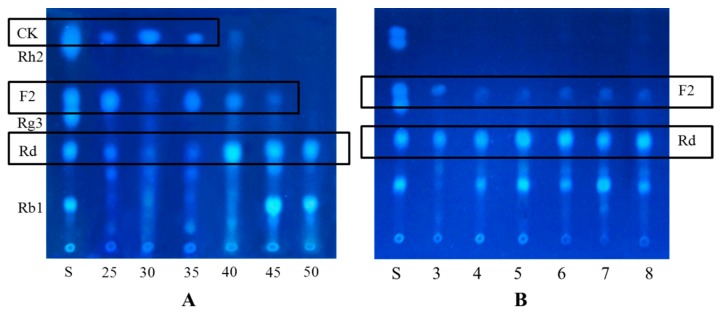
Influences of different temperature and reaction pH values on the biotransformation of Rb1. (**A**) Temperature (°C); (**B**) pH.

The optimal pH for maximal ginsenoside hydrolysis of glycosidases was in the range of 3.5–7.5. Otherwise, the activity of such enzyme decreased significantly above pH 8.0 [34,35]. In our experiments, the cultures with different pH values, being adjusted by buffer, could not positively influence the biotransformation of ginsenoside Rb1 as shown in Figure 6. *C. sinensis* might possibly suffer from malfunctioning metabolism under the pressure of osmosis.

The optimal reaction temperatures of glycosidases from the majority of bacteria, fungi and plant are in a range of 30 °C–50 °C, and these enzymes barely exhibit activity above 60 °C [36,37,38,39,40]. *C. sinensis* cultured in 25 °C–35 °C could spontaneously biotransform Rb1 into CK (3) and the optimal temperature was around 30 °C (Figure 6). The metabolism and growth of *Cordyceps sinsensis* were severely hindered above 40 °C, leading to inability in the biotransformation process. Accordingly, reaction pH and temperature were not considered in the optimization process for the efficient production of CK (3).

### 2.2. Biotransformation Pathway of Ginsenoside Rb1 by C. sinensis

**Figure 7:**
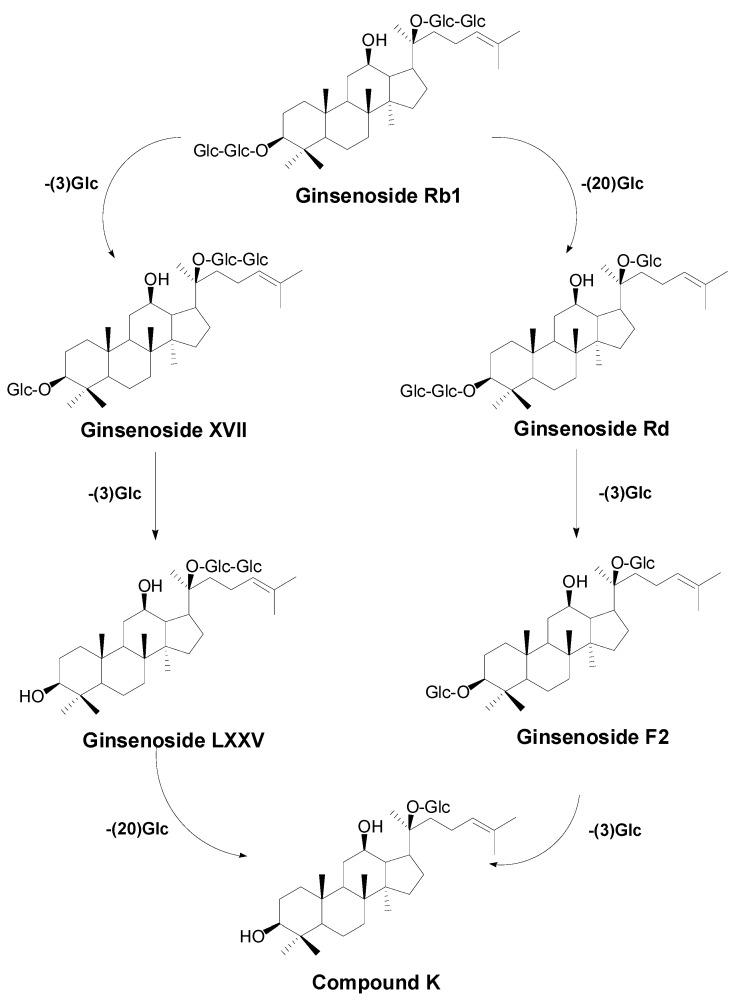
Theoretical biotransformation pathways for ginsenoside Rb1 → CK.

Two theoretical pathways for the biotransformation of Rb1 to CK could be proposed based on the structure of Rb1 (see Figure 7). One is through the intermediate Rd and F2 by hydrolyzing one glucosyl moiety at C-20 and one glucosyl moiety at C-3, respectively. The other is through ginsenosides XVII and LXXV by sequentially hydrolyzing the two glucosyl moieties at C-3. To better comprehend the biotransformation pathway for ginsenoside Rb1 by *C. sinensis*, time-course experiments were performed by HPLC-Q-TOF-MS instead of HPLC-UV to ensure the sensitivity and accuracy of metabolite analysis.

**Figure 8:**
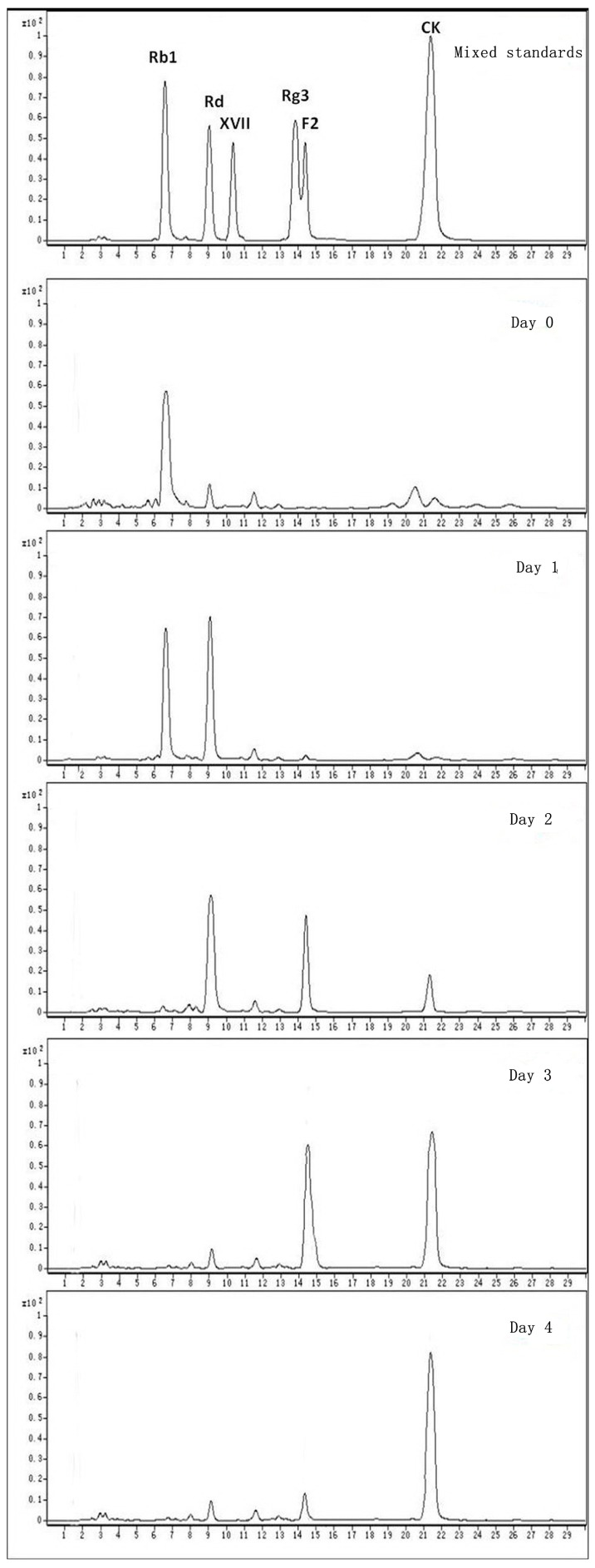
HPLC-MS analysis of the biotransformation pathway for ginsenoside Rb1.

**Figure 9:**
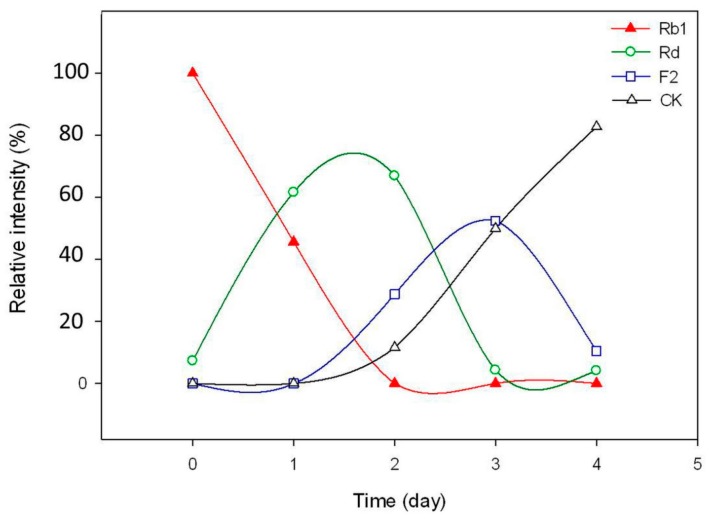
Time course for content variation of ginsenoside Rb1, Rd, F2 and CK during the biotransformation.

Ginsenoside Rb1 was gradually transformed into Rd at the 1st and 2nd day after being added to the optimal culture of *C. sinensis* and became nearly untraceable when the 2nd day was over (Figure 8 and Figure 9). Ginsenoside Rd was quickly approaching its utmost production in the first two days as long as the content of ginsenoside F2 and CK were about to accumulate from the 2nd day of biotransformation. Thereafter, the amount of Rd decreased sharply on the 3rd day, leading to a significant increase in the amount of F2 and CK. Nevertheless, most of ginsenoside F2 was ultimately converted into CK and the hydrolysis process ceased after the 4th day of biotransformation. During the whole process, neither ginsenoside XVII nor LXXV were detected, informing that the biotransformation of Rb1 to CK by *C. sinensis* was highly isomer-selective.

### 2.3. Calculation on Molar Conversion Rate of Rb1 and Purity of CK

For accurately calculating the molar conversion rate of ginsenoside Rb1 to CK, a certain amount of ginsenoside Rg1 was distributed as internal reference in the experimental cultures either before (0 day) or after (4 days) the biotransformation. Ginsenoside Rb1, Rg1, CK from both 0-day-culture and 4-days-culture were quantified by the MRM mode of HPLC-MS. The intensity of MRM peaks listed in Table 2 represented relative molar concentrations of compounds. The molar conversion rate of ginsenoside Rb1 to CK by *C. sinensis* was calculated to be approximately 82% using the equation below:

$$Molar\;conversion\;rate=\frac{MRM_{CK}/MRM_{Rg1-1}}{MRM_{Rb1}/MRM_{Rg1-2}}\times 100\%$$

*MRM_CK_*: MRM intensity of CK in the 4-days-culture; 

*MRM_Rb_*_1_: MRM intensity of Rb1 in the 0-day-culture;

*MRM_Rg_*_1–1_: MRM intensity of Rg1 in the 4-days-culture;

*MRM_Rg_*_1–2_: MRM intensity of Rg1 in the 0-days-culture

**Table 2:** MRM intensity of Rb1, Rg1, CK before and after the biotransformation.

No.	Day 0		Day 4	
	Compound	MRM Intensity (×10^7^)	Compound	MRM Intensity (×10^7^)
1	Rb1	2.3991	Rb1	-
2	Rg1	1.1094	Rg1	1.1137
3	CK	-	CK	1.9749

“-” means no detection.

**Figure 10:**
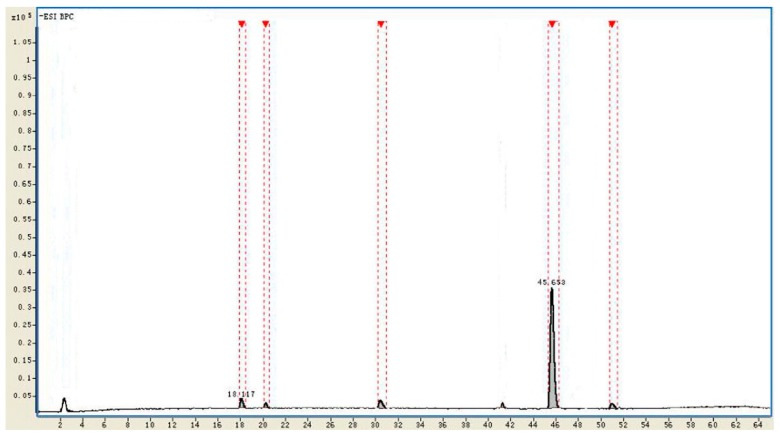
Normalization of BPC intensity by HPLC-Q-TOF-MS.

The purity of CK in the mixture of EtOAc and *n*-butanol extracts from the biotransformation culture was more than 91% by normalization of the basic peak chromatography (BPC) intensity via HPLC-Q-TOF-MS detection. The remaining Rd, F2 and other byproducts from the biotransformation of Rb1 to CK were in trace quantities, while Rb1 was untraceable (Figure 10 and Table 3).

**Table 3:** Relative concentration of ginsenoside Rd, F2 and CK after biotransformation.

No.	t_R_ (min)	Identificaiton	Intensity	Relative Intensity (%)	Diff. (ppm)
1	18.117	Rd	215,429	3.1	−1.02
2	20.185	Unkown	78,191	1.2	-
3	30.809	F2	199,501	2.8	−0.93
4	45.653	CK	6,331,023	91.4	−0.61
5	51.291	Unkown	105,419	1.5	-

In general, enzymes are superior to microorganisms in biotransformation efficiency and selectivity. In this study, however, the molar yield of CK (purity ≥ 90%) produced from the biotransformation of ginsenoside Rb1 by *C. sinensis* could reach to 82%, which was comparable to the value obtained in some cases by enzymatic biotransformation [35,41]. It is quite possible that this result would be even better after a systematic optimization by statistical methods, such as response surface methodology (RSM) and orthogonal test. Besides, the substrate selectivity of *C. sinensis* was discovered to be among the best in the biotransformation of ginsenosides by microbial systems [11,15,16,20,21]. These results demonstrated that *C. sinensis* could be a very potent biocatalyst in the production of CK.

### 2.4. Identification of the Biotransformation Metabolites

**Figure 11:**
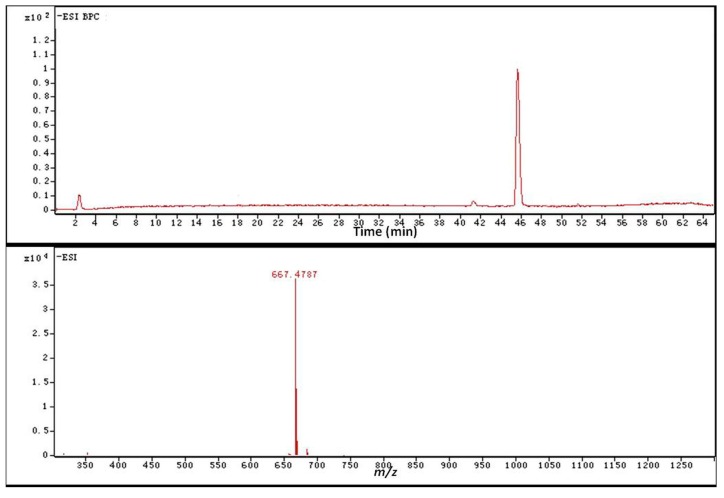
HRMS data of metabolite (3).

**Figure 12:**
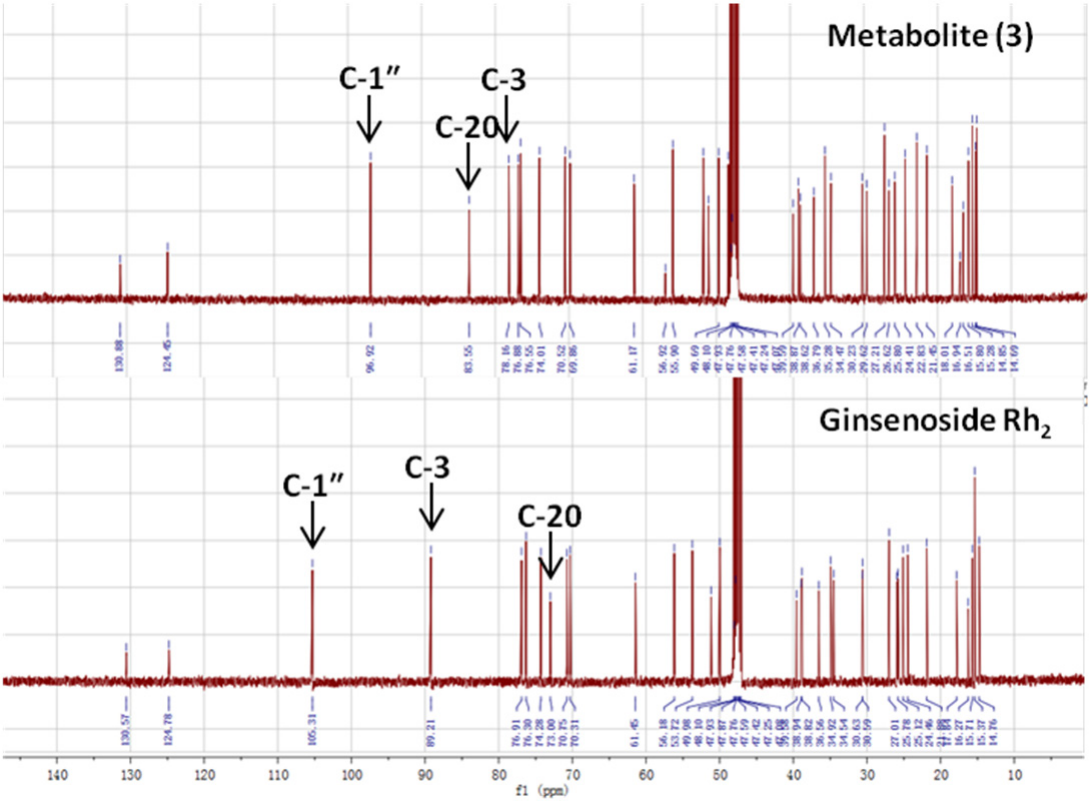
NMR differences between metabolite 3 and ginsenoside Rh2.

Metabolites 1 and 2 were identified to be ginsenoside Rd and F2, respectively, by HPLC-Q-TOF-MS and TLC comparison to reference ginsenosides, as demonstrated in Section 2.1 and Section 2.2. The combination of HPLC-Q-TOF-MS and ^13^C-NMR was used to unambiguously characterize metabolite 3. The HRMS data of 3 showed a similar [M + HCOOH − H]^−^ *m/z* with CK and Rh2 of 667.4787, calculated for a molecular formula of C_36_H_62_O_8_ (Figure 11). According to literature [42,43,44], the major structural difference between Rh2 and CK is the linking position of the glucosyl moiety, leading to obvious variations of chemical shifts at C-3, C-20 and glucosyl C-1″. As shown in Table 4 and Figure 12, metabolite 3 was proven to be CK due to the identical chemical shifts which distinguished it from Rh2 at C-3 (δ = 78.16), C-20 (δ = 83.55) and C-1″ (δ = 96.92).

**Table 4:** ^13^C-NMR chemical shifts of metabolite (3) in comparison with ginsenoside Rh2 and CK.

Carbon Site	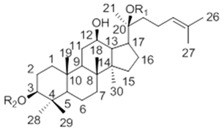		
	CK R_1_ = Glc, R_2_ = H	Metabolite (3)	Rh2 R_1_ = H, R_2_ = Glc
1	39.19	38.62	39.12
2	28.05	27.21	27.05
**3**	**77.83**	**78.16**	**88.78**
4	39.34	38.87	39.66
5	56.14	56.92	56.35
6	18.55	18.01	18.43
7	34.95	34.47	35.15
8	39.86	39.59	40.00
9	50.09	51.01	50.38
10	37.14	36.79	36.94
11	30.56	29.62	32.02
12	69.96	69.86	70.96
13	49.30	49.69	49.54
14	51.21	51.66	51.69
15	30.74	30.23	31.32
16	26.42	26.62	26.70
17	51.39	51.77	54.77
18	15.81	15.28	16.77
19	16.14	15.80	15.61
**20**	**83.08**	**83.55**	**72.94**
21	22.13	22.83	26.83
22	35.96	35.28	35.88
23	22.98	22.83	22.97
24	125.75	124.45	126.30
25	130.69	130.88	130.73
26	25.54	25.80	25.78
27	17.54	16.94	17.66
28	28.47	29.62	28.14
29	16.14	16.51	16.34
30	17.18	16.94	17.65
**Glucosyl C-1″**	**98.10**	**96.92**	**106.92**

Major differences were highlighted in bold.

## 3. Experimental Section

### 3.1. Materials and Chemicals

Reference ginsenosides Rb1, Rd, F2, Rg3, Rh2, and compound K, with purity of more than 98.0%, were purchased from National Institutes for Food and Drug Control, China. Reference ginsenoside XVII with purity of more than 95.0% was purchased from Jilin University, China. HPLC-grade methanol and acetonitrile were purchased from TEDIA (Woodstock, IL, USA). Formic acid (MS-grade) was purchased from Fisher Scientific (Far Lawn, NJ, USA). Water in the experiments was collected from a Milli-Q Ultra-pure water system (Billerica, MA, USA). Other chemicals were of reagent grade.

### 3.2. Microorganism and Culture Condition

The fungal strain *C. sinensis* (CICC14017) was purchased from the China Center of Industrial Culture Collection (Beijing, China). It had been preserved on potato dextrose agar (PDA) slant by periodical subculture. The freshly inoculated slant was incubated at 28 °C for 4 days before use. Seed culture of *C. sinensis* was prepared in Martin Broth, Modified (MBM) composing of 5 g/L peptone, 2 g/L yeast extract powder, 20 g/L d-(+)-glucose, 1 g/L K_2_HPO_4_, 0.5 g/L MgSO_4_ and 10 mg/L VB_1_. The 4-day-old culture slant was washed with sterile 0.9% NaCl solution and adjusted to approximately 10^7^ spores per mL by haemocytometer. Subsequently, the spore suspension was inoculated to MBM at a concentration of 5% (*v*/*v*) and incubated at 28 °C, 150 rpm on a thermostat shaker for 1 day before reaching its exponential growth phase. Thereafter, the 1-day-old seed culture had been kept at 4 °C until being inoculated to fermentation cultures.

### 3.3. Growth Curve of C. sinensis

The growth curve study of *C. sinensis* was conducted on basal medium (BM) composing of 10 g/L peptone and 20g/L d-(+)-glucose. One mL seed culture was inoculated into 20 mL BM as one sample before culturing in a thermostatic shaker at 28 °C and 150 rpm. Three replicates of the samples were collected and centrifuged each day to get the mycelia. Dry weights of the mycelia were calculated after being lyophilized. The growth curve of *C. sinensis* was drawn by mean values of the replicate weights in 20 days.

### 3.4. Biotransformation of Ginsenoside Rb1 by C. sinensis

The ginsenoside Rb1 biotransformation experiments were conducted under strict axenic conditions. Reference ginsenoside Rb1 was ultraviolet-disinfected and dissolved in sterilized water before being added to a 2-day-old fermentation culture of *C. sinensis* in flask. The final concentration of Rb1 in the fermentation culture was 500 μg/mL. The flask was transferred to the thermostatic shaker and fermented at 30 °C, 150 rpm for a certain number of days. One mL of fermented sample was harvested each day to detect the chemical changes of ginsenosides in the culture.

### 3.5. Sample Preparation

The collected samples were ultrasonically processed for 0.5 h before sequentially extracted by EtOAc and water-saturated *n*-BuOH three times. The EtOAc and *n*-BuOH extracts were evaporated to dryness under reduced pressure, and then dissolved and mixed in MeOH at a certain concentration. The MeOH solutions were filtrated by 0.22 μm membrane filters for TLC and HPLC-Q-TOF-MS analysis.

### 3.6. Thin Layer Chromatography Analysis

Thin layer chromatography (TLC) studies are among the key identity tests in most pharmacopoeia monographs. Although the accuracy and sensitivity of TLC is relatively lower than HPLC, it is still a robust and efficient tool for the analysis of compounds based on their different absorption characteristics.

In this study, TLC was performed with silica gel plates (GF254, Merck Millipore, Shanghai, China), and CHCl_3_–CH_3_OH–H_2_O (65:35:10, *v*/*v*, lower phase) was used as the developing solvent [45]. The spots on the TLC plates were detected through spraying with 10% H_2_SO_4_/ethanol solution, followed by heating at 105 °C for 5 min. TLC spectra were analyzed by a TLC visualizer CD 60 (DESAGA, Lindenfels, Germany) and pictures of TLC spectra were shot under UV light at 365 nm.

### 3.7. HPLC-Q-TOF-MS Analysis

The liquid chromatography separation was performed on an Agilent Poroshell 120 SB-Aq column (100 mm × 4.6 mm, 2.7 μm, 600 bar) at 30 °C. Water (0.1% formic acid) and acetonitrile (0.1% formic acid) were used as the mobile phases A and B, respectively. The gradient elution was programmed as follow: 0–5 min (5%–15% B), 5–30 min (15%–35% B), 30–40 min (35%–42% B), 40–60 min (42%–80% B), 60–65 min (80%–95% B). The flow rate was 1.0 mL/min and the injected sample volume was 10 μL.

MS detection was performed by a 6520 Q-TOF mass spectrometer (Agilent Technologies, Santa Clara, CA, USA) equipped with a dual electrospray ionization (ESI) source. The MS scan range was set at *m*/*z* 300–1300 in negative modes. The operating parameters were optimized as follow: dry gas (N_2_) flow rate, 9.0 L/min; dry gas temperature, 350 °C; nebulizer gas, 30 psi; capillary voltage, 3500 V; fragmentor, 175 V; skimmer, 65 V. We acquired MS data of the standards by flow injection analysis (FIA) in single quadrupole, product ion, single reaction monitoring (SRM), and precursor ion scan modes. The selection of collision energy for the ginsenosides involved in biotransformation pathway was made by examination of these product ion spectra. Calibrant solution A (Agilent Technologies) which contains the internal reference masses was applied to obtain high-accuracy mass measurements. Data analysis was performed on Agilent Mass Hunter Workstation software-Qualitative Analysis (version B.04.00, Build 4.0.479.5, Service Pack 3, Agilent Technologies, Inc. 2011). Molecular formulas were generated by molecular formula generator algorithm with parameter settings of C [0–80], H [0–150], O [0–60]. Other elements such as N, S, P and Cl were not considered for their rare presence in the ginsenosides [46].

### 3.8. Determination of the Metabolites

The metabolites were prepared by semi-preparation HPLC. The purified metabolites were dissolved in either methanol or CH_3_DO and analyzed via TLC, HPLC-Q-TOF-MS and NMR (BRUKER AVANCE III-500, Billerica, MA, USA), respectively.

### 3.9. Parameters Optimization for the Biotransformation of Ginsenoside Rb1

#### 3.9.1. Substrate-Feeding Time

*C. sinensis* was incubated in five flasks of BM under the condition of 30 °C and 150 rpm. The substrate ginsenoside Rb1 was distributed to the culture mediums on day 1, day 2, day 3, day 4 and day 5 at a final concentration of 500 μg/mL, respectively. After the biotransformation, the fermented samples were collected from each flask to detect the chemical changes of ginsenosides.

#### 3.9.2. Carbon Sources

Lactose, amidulin, sucrose, glucose and maltose were selected as alternative carbon sources in the BM to investigate influences on the biotransformation of ginsenoside Rb1. 10 g/L peptone was fixed as a sole nitrogen source and the concentration of these carbohydrates was 20 g/L in these experiments individually.

#### 3.9.3. Nitrogen Sources

Ten g/L of peptone, soybean powder, yeast extract, KNO_3_ and (NH_4_)_2_SO_4_ were selected as alternative nitrogen sources while 20 g/L of the optimized carbohydrate was fixed as a sole carbon source in these experiments to investigate influences on the biotransformation of ginsenoside Rb1.

#### 3.9.4. Elicitors

Eight elicitors including *p*-nitrophenyl-β-d-galactopyranoside (PNPG), sodium salicylate, MgSO_4_, ZnSO_4_, FeSO_4_, CaCl, MnSO_4_ and KH_2_PO_4_ were investigated in this study. They were all prepared in water as 10 mM solutions. All the elicitor solutions were sterilized by filtration and individually added to the fermentation cultures using the optimized carbon source and nitrogen source at a final concentration of 1 mM.

#### 3.9.5. Reaction pH

The optimization on reaction pH value was fulfilled by performing the biotransformation experiments in either citric acid–sodium citrate buffer (pH 3.0–5.0) or K_2_HPO_4_–KH_2_PO_4_ buffer (pH 6.0–8.0) containing the optimized carbon source, nitrogen source and elicitors.

#### 3.9.6. Biotransformation Temperature

The effects of temperature on the biotransformation of ginsenoside Rb1 were investigated with the optimized carbon source, nitrogen source and elicitors between 25 °C and 50 °C (25 °C, 30 °C, 35 °C, 40 °C, 45 °C, 50 °C).

### 3.10. Time-Course Experiments

Biotransformation experiments were performed under the optimized parameters (carbon source, nitrogen source, elicitors, pH and temperature) with 500 μg/L ginsenoside Rb1 for a certain number of days. Every day, 1 mL of culture medium was removed and prepared as described above. The samples were analyzed by HPLC-Q-TOF-MS. The relative concentrations of ginsenoside Rb1, Rd, F2, CK and internal reference Rg1 were determined by multiple reactions monitoring (MRM) mode with settings as follows:

(1) Rb1: *m*/*z* 1107→945 [M − H]^−^
(2) Rg1: *m*/*z* 799→637 [M − H]^−^
(3) Rd: *m*/*z* 945→783 [M − H]^−^
(4) F2: *m*/*z* 783→621 [M − H]^−^
(5) CK: *m*/*z* 621→459 [M − H]^−^

## 4. Conclusions

Although, most fungi were not comparable to soil bacteria and intestinal flora in the biotransformation of ginsenosides, we found that *C. sinensis* was highly effective and selective in the biotransformation of ginsenoside Rb1 into CK. The molar bioconversion rate of Rb1 to CK was calculated to be more than 82% and the purity of the prepared CK was over 91%. The investigation on carbon sources, nitrogen sources, elicitors, reaction pH and fermentation temperature provided substantial evidence for the discovery of potent glycosidases and the elucidation of biotransformation mechanisms. These findings should lay a solid foundation for the construction of gene-engineered strains and ultimately, the industrial preparation of CK. 
